# Effects of 200 Gy ^60^Co-γ Radiation on the Regulation of Antioxidant Enzymes, Hsp70 Genes, and Serum Molecules of *Plutella xylostella* (Linnaeus)

**DOI:** 10.3390/molecules23051011

**Published:** 2018-04-26

**Authors:** Xiaoxue Li, Lingyan Luo, Sengodan Karthi, Ke Zhang, Jianjun Luo, Qiongbo Hu, Qunfang Weng

**Affiliations:** 1 College of Agriculture, South China Agricultural University, Guangzhou 510642, China; 13580458756@163.com (X.L.); llyjay915@163.com (L.L.); karthientomology@gmail.com (S.K.); zhangke2110@163.com (K.Z.); luojianjun@scau.edu.cn (J.L.); 2 Key Laboratory of Natural Pesticide & Chemical Biology, Ministry of Education, Guangzhou 510642, China; 3 Key Laboratory of Integrated Pest Management on Crops in South China, Ministry of Agriculture, Guangzhou 510642, China; 4 Key Laboratory of Bio-Pesticide Innovation and Application of Guangdong Province, Guangzhou 510642, China

**Keywords:** diamondback moth, ^60^Co-γ radiation, antioxidant, testis

## Abstract

The diamondback moth, *Plutella xylostella* (Linnaeus), is one of the notorious pests causing substantial loses to many cruciferous vegetables across the nations. The effects of ^60^Co-γ radiation on physiology of *P. xylostella* were investigated and the results displayed that 200 Gy irradiation significantly alters the antioxidant enzyme regulation in six-day-old male pupae of *P. xylostella*. First, in our research, we detected Oxidase system and stress response mechanism of irradiated pupae, the results displayed that 200 Gy irradiation significantly alters the antioxidant enzyme regulation in six-day-old male pupae of *P. xylostella*. The levels of superoxide dismutase (SOD) and catalase (CAT) were increased significantly in contrast the level of peroxidase (POD) and glutathione S-transferase (GST) were decreased in 12–24 h post-treatment. The heat shock proteins (Hsps) gene expression level was significant increasing, maximum > 2-folds upregulation of genes were observed in peak. However, they also had a trend of gradual recovery with development. Second, we detected the testis lactate dehydrogenase (LDH) and acid phosphatase (ACP) activity found that in male adults testis they increased significantly than control during its development. Thus the present research investigation highlights that the ^60^Co-γ radiation treatments alters the physiological development of diamondback moth. The results showed that 200 Gy dosage resulted in stress damage to the body and reproductive system of the diamondback moth.

## 1. Introduction

The diamondback moth (DBM), *P. xyllostella* (L.) is one of the most critical notorious pests of cruciferous vegetables across the nations [1]. The larvae mainly feed on leaves, making holes and resulting in serious loss of vegetable production. Chemical pesticides have been used widely used to control this pest [2]. However, it has developed resistance to a series of synthetic and biological based insecticides. So there is an urgent need to develop an eco-friendly tool to reduce the global burden caused by the synthetic chemicals [3].

The sterility insect technology (SIT) is put forward by the professor A. S. Serebrovskii, its application entails mass production and then releasing sterile males to wild to mate with wild females, so as to control population [4]. The sterilized insects are exposed to ionizing radiation always from γ and x rays for SIT, whose DNA double-strand is broken randomly by radiation. DNA may be damaged by the chromosomal breaks and ROS produced in irradiation [5]. Oxidative stress plays important role in radiation response and antioxidant enzymes are the primary way to minimize stress-induced damage and regulate cell sensitivity. Studies so far have shown that γ-radiation induced Sf9 cells have higher basal APOx (4-fold), catalase (1.7-fold), and SOD (1.3-fold) activity as well as GSH levels (2.2-fold). It is suggested that there is a stronger antioxidant system in Lepidopteran insects cells to prevent radiation-induced macromolecular damage, growth inhibition, and cell death [6]. 

Radiation induces the production of reactive oxygen species [7,8]. Oxygen free radicals are a threat to aerobic organisms, whether their life expectancy is measured in days or decades. It is a crucial factor to longevity and reproduction [9,10]. During the physiological functioning of aerobic organisms, free radicals are generated and cleared; there is a balance between these two processes, and if the balance is disrupted, it will result in damage and bodily lesions. Insects are not immune to the ravages of reactive oxygen species (ROS), which are a by-product of oxidative metabolism in aerobic cells and are produced following exposure of cells and tissues to various stressors [11,12,13]. Low levels of ROS are generally considered to be harmless to cells and may even perform useful functions. The endogenous enzymatic antioxidant system is important for protecting the organism against high levels of ROS. This system is mainly composed of the enzymes superoxide dismutase (SOD), catalase (CAT), peroxidase (POD), and glutathione S-transferase (GST) [14,15,16,17]. 

Ionizing radiation produces ROS, which can induce the expression of HSP. In return, HSP can also play a role in protecting cell tissues from injury by inhibiting ROS [18]. Insects respond to high temperatures and rapid increases in various chemical and physical stresses through the deployment of a group of endogenous proteins collectively referred to as ‘heat shock proteins’ or HSPs [19]. The Hsp70 family is one of the most abundant Hsp families and is highly conserved. These proteins are characterized by the upsurge levels of transcription and are the most sensitive to various deleterious stimuli [20]. Hsp70 is expressed at low basal levels under non-stress conditions but can be quickly induced by heat shock and other environmental stresses [21,22].

Based on the previous study, it is well known that the effect of irradiation on the reproductive system of insects is more obvious [4]. Lactate dehydrogenase (LDH) is the key enzyme in Embden–Meyerhof–Parnas pathway, and its activity changes directly affects the energy providing of cell. Acid phosphatase (ACP) is a hydrolase that catalyzes the hydrolysis of phosphoric acid to produce inorganic phosphoric acid under acidic conditions related to the absorption and transport of nutrients. LDH and ACP were used as a marker enzyme in the testes of *Bufo Gargarizans* intaking heavy metal [23]. LDH enzyme is a vital source of producing energy in glucose metabolism in spermatocyte [24]. The ACP enzyme is mainly used as a biomarker enzyme to detect the incidence of spermatogenic disorders [25].

In this article, the present investigation was aimed to detect the activity of oxidase and Hsp70s heat shock protein in the parental pupa of *P. xylostella.* As reported, a dose of 200 Gy gamma radiation for *P. xylostella* was a substerilizing dose, inducing 26% and 36% sterilities respectively [26]. This enabled further exploration of the effect of irradiation on testis. Moreover, we determined the LDH and ACP activity in the testis of pupae and adult of irradiated *P. xylostella.* The research on sperm is being carried out in the laboratory but is not yet completed. 

## 2. Results and Discussion

### 2.1. Effect of ^60^Co-γ Radiation on the Antioxidant Enzymes of Pupae Irradiated with 200 Gy

The SOD and CAT level was significantly higher from 12 h to 72 h in comparable to control, with the most significant difference being observed at 24 h. However, POD and GST levels were prominent 48 h after irradiation. Both enzymes showed a trend of increasing, reaching a peak at 24 h, followed by a gradual decrease. 

**Figure 1:**
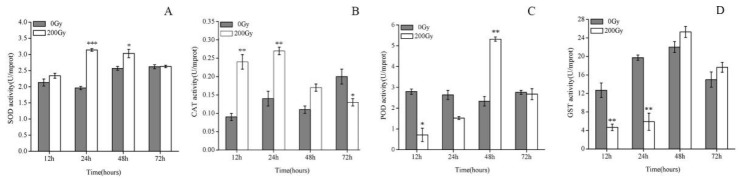
Effects of ^60^Co-γ irradiation on activity of (**A**) SOD, (**B**) CAT, (**C**) POD, (**D**) GST in the pupal stage of *P. xylostella* at different time points post irradiation. Values are the mean ±SD (*n* = 3). Asterisk designates statistically significant difference between control and irradiated pupae (*** *p* < 0.001; ** *p* < 0.01; * *p* < 0.05).

SOD activity was significantly increased at 24 h and 48 h after irradiation than control and reduced to normal, in significant as compared to control, by 72 h (Figure 1A). Similarly, the CAT activity at 12 h and 24 h was significantly increased compared with control, while the activity was not prominent at 48 h and 72 h becoming decreased with control (Figure 1B). Correspondingly, the POD activity at 48 h displayed higher significant rate as compared to other measurement times and control (Figure 1C). In contrast, the GST activity at 12 h and 24 h post measurement times was significantly lower than 48 h and 72 h post-irradiation, and the control (Figure 1D). 

SOD is an antioxidant protein that plays an important role in reducing high levels of intracellular superoxide radicals induced by extracellular stimuli such as ^60^Co-γ irradiation. In the present study, the observed changes in SOD activity indicated that ^60^Co-γ irradiation induced the production of superoxide free radicals in diamondback moth pupae. The antioxidant defense systems of two lepidopteran insect cell lines have been reported [14]. SOD activity gradually increased from 12 h to 24 h after the insects were exposed to irradiation, and the reduced to the level of control after 72 h, suggesting that SOD was stimulated by scavenging superoxide radicals to protect the pupae from radiation stress, and its activity subsequently returned to normal levels after 72 h. It has been found that high doses of UV irradiation suppress the activity of protective enzymes, such as SOD, in normal cells [27]. This finding is consistent with previous reports showing that CAT can protect against oxidative stress and extend the life of insects [28,29]. This CAT activity was still sufficient to cope with the excess H_2_O_2_ induced by irradiation stress. Previous studies have shown that enzyme activity can be reduced via negative feedback from excessive substrate levels or damage by oxidative modification [30]. The significant increase in SOD and CAT activity observed in response to ^60^Co-γ irradiation and the simultaneous decrease in POD activity suggested that CAT may play a more important role in scavenging H_2_O_2_ than POD [31]. GST effectively metabolizes lipid peroxides and can be considered the main antioxidant enzyme in insects [32]. These results suggest that with the prolongation of recovery time, the activity of SOD, CAT, POD, GST decreased, the antioxidant capacity decreased, and the level of oxidative stress increased. 

### 2.2. Effect of ^60^Co-γ Radiation on the Expression of Hsp70s Genes of Pupae Irradiated with 200 Gy

**Figure 2:**
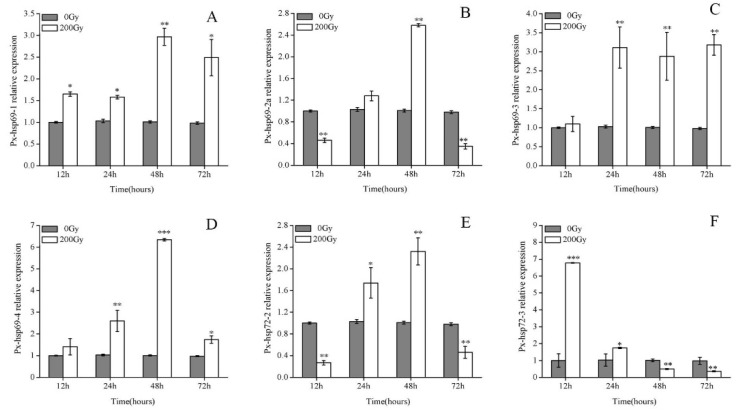
Effect of ^60^Co-γ radiation stress on the expression of (**A**) Px-hsp69-1, (**B**) Px-hsp69-2a, (**C**) Px-hsp69-3, (**D**) Px-hsp69-4, (**E**) Px-hsp72-2, (**F**) Px-hsp72-3 in DBM pupae. Values are the mean ±SD (*n* = 3). Asterisk designates statistically significant difference between control and irradiated pupae (*** *p* < 0.001; ** *p* < 0.01; * *p* < 0.05).

The basal mRNA expression level of the six px-hsp70s was significantly altered at different time between 0 Gy and 200 Gy (Figure 2). The basal relative mRNA expression levels of Px-hsp69-1, Px-hsp69-3, and Px-hsp69-4 showed upregulation. The Px-hsp69-1 displayed higher expression rates from 12 h to 72 h, reaching a peak of approximately three-fold at 48 h. However, Px-hsp69-3 level was not substantially regulated at 12 h but the expression was upregulated three-fold higher from 24 h to 72 h. Correspondingly, Px-hsp69-4 displayed no significant upregulation at 12 h and 72 h but displayed prominent expression level at 48 h. Similarly, the expression levels of Px-hsp69-2a and Px-hsp72-2 were prominent at 48 h as compared to the levels at other time intervals. The basal relative mRNA expression level of Px-hsp72-3 showed a seven-fold higher expression rate at 12 h and it was significantly different from the expression rates at 24, 48, and 72 h after irradiation. 

A common physiological response of organisms to environmental stress is to upregulate the expression of Hsp genes, particularly those from the Hsp70 family, which are believed to help the organism cope with various potential disadvantages by refolding damaged proteins, preventing denaturation of proteins due to aggregation and promoting the transport of proteins to intracellular locations for degradation [33,34]. In the present study, the expression level of Px-hsp70s was upregulated in pupae under irradiation stress. This phenomenon has been observed in almost all organisms tested to date [35,36,37,38], which indicates that inducible Hsp70s play a critical role in cell protection and the enhancement of irradiation tolerance. Under the stress of irradiation, the expression of HSPs is induced in cells through changes in gene expression, thus initiating the endogenous protection mechanism. However, the expression levels of Px-hsp69-2a, Px-hsp72-2, and Px-hsp72-3 were downregulated at 72 h, suggesting that the irradiated insects had adapted to irradiation stress following hsp70 upregulation. Likewise, the adaptive response to ionizing radiation is a phenomenon in which the upregulation of Hsp70s can mitigate the deleterious effects of exposure to ionizing radiation [39]. Hsp70s are involved in the process of irradiation adaptation, likely due to the production of ROS. ROS can induce Hsp70s expression, and in turn, Hsp70s can protect cells and tissues from damage by inhibiting ROS [40,41]. Hsp70 is mainly involved in protein folding, assembly, transport, cell protection, antigen presentation, and tumor immunity. It can restore or accelerate the removal of denatured proteins and stabilize cell structure to achieve thermal tolerance, which plays an important role in the organism [42]. The current results illustrate that the external stress through irradiation on pupae increased the Hsp70s expression pattern for initiating the endogenous protection mechanism [37,43]. Previous research suggests that genes encoding Hsp70s displayed higher expression levels when the insects were subjected to any external stress or stimuli. Insects can resist their external stress by increasing Hsps expression [39]. Maybe the *Plutella xylostella* has radiation resistance to 200 Gy irradiation doses.

### 2.3. Effects of ^60^Co-γ Radiation on LDH and ACP Activity of Testes Irradiated with 200 Gy

**Figure 3:**
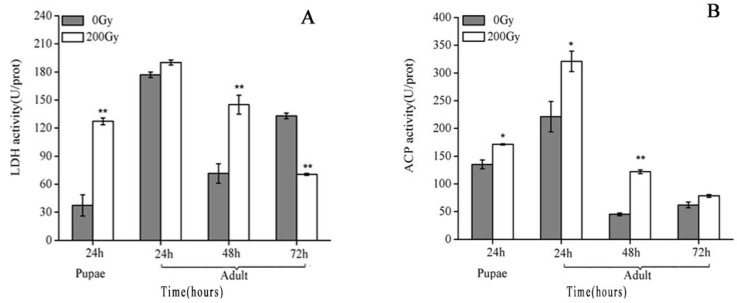
Effects of ^60^Co-γ irradiation on activity of (**A**) LDH and (**B**) ACP in *Plutella xylostella* testis. Values are the mean ± SD (*n* = 3). Asterisk designates statistically significant difference between control and irradiated males. (** *p* < 0.01; * *p* < 0.05).

A dose of 200 Gy gamma radiation induced LDH and ACP increased (Figure 3). LDH capacity was increased by more than three times in the pupae testis, decreasing significantly 72 h after emergence (Figure 3A). ACP activity remained substantially higher than the control but recovered to control levels 72 h after emergence (Figure 3B).

LDH and ACP are common indicators indicating tissue damage produced by chemical stimulation in the testis of fish [44]. In an experiment of selenium inducing reproductive damage of the male rat testis, the activity of LDH and ACP enzyme increased significantly [45]. The study found that 900 MHz electromagnetic irradiation damage the reproductive system of rats, inducing the decrease of sperm count, deformity of sperm, and the increase of LDH enzyme activity [36]. In our results, the activity of LDH and ACP was significantly higher than control and reach the peak at 24 h after emergence. A mating trial of *P. xylostella* complete at first and second day post emergence and the female fertility, fecundity, and egg fertility was strongest than other day [46]. This suggested that a significant increase of LDH and ACP activity may be associated with testes damage.

## 3. Experimental

### 3.1. Insects

The pupae of *P. xyllostella* were collected from a cabbage (*Brassica parachinensis Bail*) mustard field in Guangdong Province, China. They were reared and maintained under laboratory conditions at 25 ± 1 °C, 60~70% RH, and 8:16 h L:D. The resulting moths were confined in a cage (50 cm in length, 45 cm in width, 45 cm in height) with 10% honey for feeding and allowed to mate. Cabbage (*Brassica parachinensis Bail*) seedlings (three days) were placed in the tray for egg laying and also for larvae to feed (25 ± 1 °C 60~70% RH, and 8:16 h L:D). 

### 3.2. Irradiation

Irradiation was applied with γ rays from ^60^Co-γ irradiation source designed by Nordion (Ottawa, ON, Canada). The ^60^Co–γ radiation source was purchased from Furui High-Energy Technology Co. Ltd. (Nansha District, Guangzhou, Guangdong Province, China). The treatment dosage rate was 16.67 Gy/min and the dosage rate was measured using a Fricke dosimeter adapted on ISO/ASTM E 1026-04. The six-day-old pupae were chosen (due to radiation resistant and convenient for in vitro experiments) and were transferred into a culture dish and exposed to ^60^Co–γ ray irradiated for 12 min.

### 3.3. Enzyme Assay

A total of 15 pupae per treatment were randomly selected and subjected to ^60^Co–γ treatment irradiation at regular intervals of 12, 24, 48, and 72 h. After treatment, the samples were immediately frozen in liquid nitrogen and stored at −80 °C prior to analysis. The controls were subjected to the same conditions but were not irradiated. For each treatment, three replicates were performed.

The activities of SOD, CAT, POD, and GST were determined spectrophotometrically according to the manufacturer’s protocol, with a number of modifications, using commercially available assay kits (Nanjing Jiancheng Bioengineering Institute, Jiangsu, China). SOD activity was measured at 550 nm using the xanthine and xanthine oxidase systems. CAT activity was determined by measuring the decrease in absorbance at 240 nm for H_2_O_2_ decomposition. Whereas POD activity was determined at 420 nm via catalytic oxidation in the presence of H_2_O_2_ and the substrate. GST activity was determined using 1-chloro-2,4-dinitrobenzene (CDNB) as substrate. The formation of the GSH-CDNB conjugate was monitored by the change in absorbance at 412 nm. 

### 3.4. Real-Time Quantitative PCR (qPCR) Analysis of Hsp70s Genes

Whole genomic RNA was extracted and first-strand cDNAs were synthesized, and real-time quantitative PCR (qPCR) analysis of Hsp70s was performed according to the manufacturer’s instructions for the RNA Simple Total RNA Extraction Kit, the FastQuant RT Kit, and the SuperReal PreMix Plus Kit, respectively (Tiangen Biotech Co., Ltd., Beijing, China). The primers for qPCR analysis of the Px-hsp70s were synthesized in the company (Sangon biological engineering Co., ltd., Shanghai, China) and primers for β-actin (housekeeping gene) were used as an endogenous control [38]. qPCR was performed with a SuperReal PreMix Plus (SYBR Green) Kit according to the following program: 95 °C for 15 min, followed by 40 cycles of 95 °C for 10 s and 55 °C for 30 s, with plate reading for 32 s. Subsequently, the homogeneity of the PCR product was confirmed by melting curve analysis. The expression level of each gene was calculated according to the cycle threshold (Ct) equation and the standard curve. Therefore, the normalized expression value for the target gene was calculated by comparing the expression value for the target gene with the expression value for β-actin [47,48].

### 3.5. Dissection of Pupae and Males

A total of 200 testes was dissected and used for each treatment. The testes of irradiated pupae (24 h after irradiation) whose testes were more complete and males (24 h after irradiated pupae emergence) which was always 48 h after treatment. The pupae were dissected within 20 µL PBS on the glass slices directly under the stereoscope (Motic SMZ-171), and the adult males were killed in alcohol for 10 s and then dissected under the stereoscope within 20 µL PBS. The testes were immediately placed in liquid nitrogen stored at −80 °C or in fixed liquid for follow-up tests [49].

### 3.6. LDH and ACP Analysis

The testes prepared at −80 °C were used to in this experiment. The activities of lactate dehydrogenase (LDH) and acid phosphatase (ACP) were detected according to the instruction of matched test kit (Nan Jing Jian Cheng Bioengineering Institute). The absorbance of LDH and ACP was performed using microplate technique. The activities of LDH and ACP were detected at 450 nm and 520 nm respectively. 

### 3.7. Statistical Analysis

Statistical data from the experiments were subjected to analysis of variance (ANOVA) using SPSS software version 18.0 (SPSS, Inc., Chicago, IL, USA). The effect of different times on enzyme activity was analyzed by linear discriminant analysis (LDA). A value of *p* < 0.05 was considered significant.

## 4. Conclusions

As reported, mild stimulation of the cell can increase the activity of antioxidant enzymes and reduce the damage caused by stress factors such as radiation [50]. In our research, a 200 Gy dose of ^60^Co-γ irradiation produced ROS and then induced oxidative stress, upregulating the expression levels of Px-hsp70s, disrupting the functional activity of proteins, and enhancing the activity of the protein oxidation process. A 200 Gy dose of ^60^Co-γ irradiation had an effect on the testis. The changes of these indexes reduced the activity to the control level with the growth of the organism. *Plutella xylostella* may have a certain recovery ability after 200 Gy irradiation treatment. Sub-lethal stresses caused physiological changes that induced greater longevity and stress resistance [51,52].
